# Transcriptional profiling analysis providing insights into desiccation tolerance mechanisms of the desert moss *Syntrichia caninervis*

**DOI:** 10.3389/fpls.2023.1127541

**Published:** 2023-02-23

**Authors:** Ruirui Yang, Xiaoshuang Li, Qilin Yang, Mingqi Zhao, Wenwan Bai, Yuqing Liang, Xiujin Liu, Bei Gao, Daoyuan Zhang

**Affiliations:** ^1^ State Key Laboratory of Desert and Oasis Ecology, Key Laboratory of Ecological Safety and Sustainable Development in Arid Lands, Xinjiang Institute of Ecology and Geography, Chinese Academy of Sciences, Urumqi, China; ^2^ College of Resources and Environment, University of Chinese Academy of Sciences, Beijing, China; ^3^ Xinjiang Key Lab of Conservation and Utilization of Plant Gene Resources, Xinjiang Institute of Ecology and Geography, Chinese Academy of Sciences, Urumqi, China; ^4^ Turpan Eremophytes Botanical Garden, Chinese Academy of Sciences, Turpan, China

**Keywords:** *Syntrichia caninervis*, desiccation tolerance, phenylpropanoid biosynthesis, alpha-linolenic acid, photosynthesis, transcription factor, transcriptome

## Abstract

*Syntrichia caninervis* is a desiccation tolerant moss and is the dominant bryophyte found in biological soil crusts in the Gurbantunggut desert. In this study, we assessed the transcriptome profiles of *S. caninervis* gametophytes during the dehydration-rehydration (D-R) process (across 9 time points) using Illumina sequencing. In total, 22489 transcripts were identified, including 5337 novel transcripts, that mapped to the reference genome. A total of 12548 transcripts exhibited significant alterations in the D-R samples compared with the control samples. The differentially expressed transcripts (DETs) possessed several enriched Gene Ontology terms, such as “water stress response”, “oxidation-reduction process”, “membrane metabolism”, “photosynthesis”, and “transcription factor activity”. Moreover, during early dehydration stress, the DETs were significantly enriched in stress-related pathways from the Kyoto Encyclopedia of Genes and Genomes, such as “phenylpropanoid biosynthesis”, “alpha-linolenic acid metabolism”, and “fructose and mannose metabolism”. Photosynthesis-related transcripts (e.g., *ScPsa H*, *ScRubisco*, and *ScLhcb1*) were inhibited during the dehydration treatment and significantly accumulated during the late rehydration period. Most transcripts from the late embryogenesis abundant proteins (*LEA*) and early light-inducible protein (*ELIP*) families strongly accumulated at the late dehydration stage. These pathways were positively correlated with the content changes of absolute water content and *Fv/Fm* values, alongside peroxidase and superoxide dismutase activities. Seven transcription factor families, including *AP2-ERF*, *bHLH*, *G2-like*, *MYB, NAC*, *WRKY*, and *bZIP*, were enriched in DETs during D-R treatment. This study is the first transcriptome analysis using the *S. caninervis* genome for gene annotation and multigroup D-R treatment points. Our results demonstrated the detailed dynamic changes in the transcriptome of *S. caninervis* during the D-R process. These results also improve understanding of desiccation tolerant plants’ adaptations to desiccation stress at the transcription level and provide promising gene resources for transgenic crop breeding.

## Introduction

*Syntrichia caninervis* is a moss with extremely vegetative desiccation tolerance ability. This moss is a dominant component of biological soil crusts in the Gurbantunggut Desert of China (Zhang, 2005). It is frequently exposed to desiccation and the extreme high/low temperatures of the desert (Silva et al., 2021). *S. caninervis* has been classified as category “A” for desiccation tolerance (DT) (Wood, 2007), thereby exhibiting strong cell protection and repair adaptability with DT regulation at a morphological (Pan et al., 2016), physiological, biochemical (Wu et al., 2009; Yin et al., 2017), and molecular level (Li et al., 2016; Liang et al., 2017; Liu et al., 2020; Li et al., 2023). Many genes isolated from *S. caninervis* have been observed to enhance abiotic and biotic stress resistance in model plants. Additionally, some of these genes have been successfully used to create stress-tolerant cotton, tobacco, and *Medicago sativa* plants (Yang et al., 2015; Yang et al., 2016; Yang et al., 2021; Zhang et al., 2022). For example, *ScALDH21* overexpression enhanced drought and salt tolerance in transgenic *Arabidopsis*, tobacco, and cotton plants, and others (Yang et al., 2012; Yang et al., 2015; Yang et al., 2016; Wang et al., 2020; Yang et al., 2021). Furthermore, *ScDREBs* and *ScABI3* confer osmotic and salt tolerance to *Arabidopsis* (Li et al., 2016; Liang et al., 2017; Li et al., 2017; Zhang et al., 2018; Li et al., 2019a; Liu et al., 2022). Additionally, *ScAPD1-like* enhanced resistance to *Verticillium* wilt in transgenic *S. caninervis* and *Arabidopsis* (Li et al., 2023). Finally, *ScELIPs* have been observed to improve the photosynthetic apparatus protection of transgenic *Arabidopsis* under high light stress (Liu et al., 2020).

Transcriptomes of plant responses to dehydration-rehydration (D-R) have been established for several DT plants, including angiosperms, ferns, and bryophyte species. Specifically, the transcriptomes related to DT bryophytes include *Marchantia inflexa* (Marks et al., 2021), *Marchantia polymorpha* (Godinez-Vidal et al., 2020), *Physcomitrella patens* (Hiss et al., 2014), *Tortula ruralis* (Oliver et al., 2004; Oliver et al., 2009), *Bryum argenteum* (Gao et al., 2017), and *Grimmia pilifera* (Liu et al., 2022). However, most of these bryophyte transcriptomes were annotated using information from other species, e.g., *P. patens*, rather than their own genomes. Additionally, very little transcriptome data are available for DT moss. Previous transcriptomic analyses have been conducted on *S. caninervis* (Gao et al., 2014). However, this analysis of *S. caninervis* has only been conducted with a mixed transcriptome of diverse moss samples collected under D-R treatment and the dynamic transcriptional changes occurring during the D-R processes were not evaluated. Moreover, gene annotation of this transcriptome has been performed using the genome of *P. patens*. Recently, the *S. caninervis* genome has been sequenced at the chromosomal level (Silva et al., 2021), thereby providing a robust model for studies concerning plant DT mechanisms, including transcriptional regulation, and an excellent source of novel stress related genes.

Although many gene functions of *S. caninervis* have been studied, there is little information regarding the DT mechanism of *S. caninervis* at the integral transcriptional level in *S. caninervis* during the D-R process. In this study, a series of temporal transcriptome profiles of *S. caninervis*, involving nine D-R treatment time points, were investigated using the *S. caninervis* genome for gene annotation. Our results found several key pathways and critical transcription factors (TFs) were modified in response to desiccation stress in *S. caninervis*. We also provided a detailed picture of the dynamic changes of the *S. caninervis* transcript during the D-R process, improved understanding of plant adaptations to DT, as well as providing promising candidate gene resources for crop stress tolerance breeding.

## Materials and methods

### Plant material and dehydration-rehydration treatment

Dry *S. caninervis* samples were collected from the Gurbantunggut Desert in Xinjiang, Northwest China (Fukang County, 44°32′30″N, 88°6′42″E). The collected wild moss samples were stored at 25°C under dark conditions. The gametophytes of these samples were completely desiccated and kept in a dormant state. For experiments, the dried wild gametophytes were, first, fully hydrated with ultrapure water for 24 h; then, the slow-dried method was used to keep *S. caninervis* samples at a relative humidity (RH) of 66.67% (-57 MPa) at 25°C, as described previously (Liang et al., 2021). Samples were collected after 0, 2, 6, and 24 h of dehydration (0 h as the control). Early dehydration occurred at 2 and 6 h (D2h and D6h), and late dehydration occurred at 24 h (D24h). The dehydrated samples were subsequently rehydrated by transferring the dehydrated gametophytes to new Petri dishes at 25°C with filter paper that was saturated with ultrapure water. Rehydrated samples were then harvested at 0.5, 2, 6, 24, and 48 h. Early rehydration was considered to be 0.5, and 2 h (R0.5h and R2h), whereas late rehydration times were 6, 24, and 48 h (R6h, R24h, and R48h, respectively). Fully rehydrated samples without dehydration (0 h) served as the reference control. All samples were frozen in liquid nitrogen immediately after harvest and stored at -80°C. The prepared samples were subjected to transcriptome sequencing and physiological analysis. Photographic records of *S. caninveris* phenotypes, in addition to the measurement of the absolute water content (AWC) and *Fv/Fm* (optimal/maximal photochemical efficiency of PSII) were performed at each treatment time point.

### Physiological indicator measurements

AWC was measured in gametophytes of *S. caninervis* at various time points during the D-R process. AWC curves, plotting the water content on a dry weight basis, were produced using the following formula (Rathnayake et al., 2019): AWC (g g^-1^ DW) = (FW-DW)/DW; FW indicates the fresh weight measured at every time point during the D-R process, and DW indicates the weight measured after drying for 48 h at 80°C in an oven.

*Fv/Fm* was measured using a portable modulated fluorometer (PAM-2500; Heinz, Walz, Germany). The saturation pulse method was used to calculate *Fv/Fm*, which was measured in the dark after the box was covered for > 30 min. Parameter settings were based on the recommendations of Zhang et al. (2011).

The activities of the physiological indicators H_2_O_2_, malondialdehyde (MDA), peroxidase (POD), and superoxide dismutase (SOD) were measured using detection assay kits (Nanjing Jiancheng Bioengineering Institute, Nanjing, China), according to the manufacturer’s instructions.

### Library construction and transcriptome sequencing

A total of 27 samples, consisting of 3 biological replicates from 9 time points of D-R treatment (0 h, D2h, D6h, D24h, R0.5h, R2h, R6h, R24h, R48h) were used for the transcriptome analysis. Illumina sequencing was performed separately for these 27 samples, which was combined with UMI-RNA sequencing technology by the Novogene Company (Beijing, China). First, 1 µg of RNA was used as input material per sample for RNA sample preparation. Briefly, mRNA was purified from the total RNA using poly T oligo-attached magnetic beads. First-strand cDNA was synthesised using random hexamer primers and RNase H (M0297, NEB, Beijing) at 37°C in first-strand synthesis reaction buffer (E7530, NEB, Beijing). Later, the cDNA library fragments were purified with the AMPure XP system (Beckman Coulter, Beverly, USA) to select cDNA fragments of 100-200 bp in length. Adapter ligation was conducted at 25°C for 10 min and performed prior to PCR. Then, PCR was performed using the Phusion High Fidelity DNA polymerase(M0530S, NEB, Beijing). Finally, the transcriptome was sequenced using an Agilent Bioanalyzer 2100 system platform (Agilent Bioanalyzer, Germany).

### Quantification of transcript abundance and identification of differentially expressed transcripts

Qualified clean reads were mapped to the *S. caninervis* genome sequence (Silva et al., 2021) using HISAT v2.0.4 software (Kim et al., 2015). The genome positioning results of all sequenced reads were assembled into transcripts using the Cufflinks software. HTSeq v0.9.1 (Putri et al., 2022) was used to count the read numbers mapped to each gene. Next, fragments per kilobase of exon model per million mapped fragments (FPKM) of each transcript were calculated based on the length of the transcript; then, the read count was mapped to this transcript (Trapnell et al., 2010). Differentially abundant transcripts between the two experimental groups were analysed using the DESeq R package (1.18.0) (Wang et al., 2010). DETs were screened from transcriptome data; *p* ≤ 0.05, and Log_2_ fold change ≥ 1 were set as the thresholds for significantly differential abundance. Heatmap analysis of the transcript abundance patterns and circos of the transcriptome was performed using TBtools software (Chen et al., 2020).

### Abundance patterns of differential transcripts during D-R using GO, KEGG, and WGCNA-trait analysis

Cluster analysis of the transcript abundance patterns was performed using the TCseq R package using the K-means method. In K-means clustering, the FPKM value of the transcript must first be Z-score standardised. Gene Ontology (GO) enrichment analysis of DETs was conducted using the GOseq R package with significant enrichment being defined by a *p*-value ≤ 0.05.

Pathway enrichment analysis was performed using the Kyoto Encyclopedia of Genes and Genomes (KEGG) database (Kanehisa et al., 2007) (http://www.genome.jp/kegg). We used KOBAS software (Mao et al., 2005) to test the statistical enrichment of DETs in KEGG pathways. Pathways with *p* ≤ 0.05 were considered to be significant. Additionally, weighted correlation network analysis-trait (WGCNA-trait) analysis (Langfelder and Horvath, 2008) was conducted using the BMKcloud platform (www.biocloud.net).

### RT-qPCR analysis of the expression of desiccation tolerance-related transcripts

RT-qPCR was performed to verify the transcriptome data of the selected DETs. Total RNA was extracted using a Plant RNA Kit (Omega Bio-Tek, Guangzhou, China). First-strand cDNA was synthesised with 1 μg of total RNA using the PrimeScript RT reagent Kit with gDNA Eraser (RR047A, Takara, Japan). For RT-qPCR, the TB Green TM Premix Ex Taq TM II kit (TaKaRa, Dalian, China) was used to produce 20 μL reactions consisting of 2 μL cDNA (50 ng/L), 0.5 μL forward primer (10 μmol/L), 0.5 μL reverse primer (10 μmol/L), 10 μL TB Green Premix Ex Taq II (2×) (RR820Q, Takara, Japan) and 7 μL ddH_2_O. For PCR, denaturation was conducted at 95°C for 30 s, followed by 40 cycles of the following conditions: denaturation at 95°C for 5 s, annealing at 60°C for 30 s, and final extension step at 95°C for 15 s. RT-qPCR was performed using a CFX96 Real-Time PCR System (Bio-Rad, Hercules, CA, USA). *ScTubulin* was used as the reference gene for normalisation (Li et al., 2015). Relative abundance levels were calculated using the 2^-ΔΔCt^ method (Livak and Schmittgen, 2001), with three biological replicates and three technical replicates. Primers used for RT-qPCR were designed using the NCBI primer BLAST program and are listed in **Supplementary Table S1**.

### Statistical analysis

Statistical analyses were performed using the GraphPad Prism 9 software for Windows (version 9.0.0, 2020). All data were analysed using an analysis of variance (ANOVA) test at a 95% confidence level. Significant differences were determined using Fisher’s least significant difference multiple comparison test. The data shown are as the mean ± standard deviation (SD) of three replicates; the significance level relative to the controls was assessed at *p < 0.05, **p < 0.01, and ***p < 0.001. Column graphs were generated using GraphPad Prism 9 software. Adobe Photoshop CC 2019 was used for image processing.

## Results

### Changes in phenotype and physiological parameters during the D-R process

**Figure 1:**
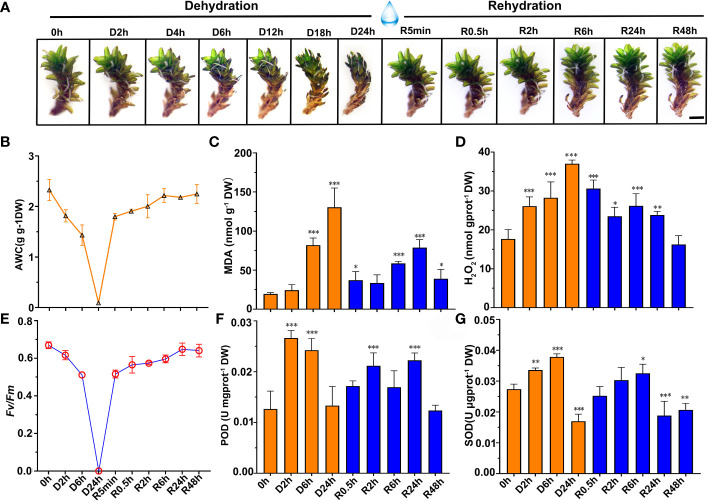
Phenotype, physiological parameters change of *S. caninervis* under D-R treatments. **(A)** Phenotype of *S. caninervis* under D-R conditions, scale bars: 2 mm; **(B)** AWC; **(C)** *Fv/Fm*; **(D)** MDA content; **(E)** H_2_O_2_ content; **(F)** POD activity and **(G)** SOD activity; treatments during 24 h of desiccation (D h) and 48 h of rehydration (R h); data represents the mean ± SD from six biological replicates.*p < 0.05, **p < 0.01, ***p < 0.001, Fisher’s least significant difference test.

In a closed atmosphere at 67% RH for dehydration treatment, the gametophytes of *S. caninervis* curled and shrunk. These gametophytes completely dried and equilibrated osmotic potential with the surrounding air after 24 h. Once rehydrated, the gametophytes quickly recovered to their initial states (**Figure 1A**). Prior to desiccation (0 h), the mean AWC of *S. caninervis* gametophytes was 2.33 g g^-1^ DW (**Figure 1B** and **Table S2**). This steadily declined to a stable AWC of approximately 0.045 g g^-1^ DW at D24h. Overall, these gametophytes exhibited a loss of approximately 98.06% of bulk water in 24 h of dehydration, at which point they were considered to be desiccated. Upon rehydration, desiccated gametophytes recovered fresh weight rapidly, to 77% of the control AWC within 5 min. Full rehydration occurred 6 h after the addition of water to desiccated gametophytes (**Figure 1B** and **Table S2**). *Fv/Fm* values decreased as dehydration progressed; after 2 h and 6 h of dehydration, *Fv/Fm* values decreased by 7.89% and 23.59%, respectively, compared to the control levels (**Figure 1C** and **Table S2**). As desiccation reached 24 h, the *Fv/Fm* declined to values close to zero. Nonetheless, *Fv/Fm* recovered rapidly to 77.10% of the control value within 5 min of rehydration, and continued to recover, approaching control levels by 24 h of rehydration (**Figure 1C** and **Table S2**).

To better quantify the oxidative status of the gametophytes, the contents of MDA, a marker of lipid peroxidation and membrane damage, and H_2_O_2_ were measured in gametophytes during the D-R process (**Figures 1D, E**). Our results demonstrated that MDA content exhibited a biphasic response to the D-R process, peaking at 24 h of dehydration, increasing to 133.79 ± 22.09 nmol g^-1^ DW, followed by a decline to a minimum at 0.5 h of rehydration; a second peak was, then, observed at 24 h of rehydration and recovered to the control (0 h) at 48 h (**Figure 1D**). The MDA content, like H_2_O_2_, reached a peak value (36.9 9 ± 2.00 nmol gprot^-1^ DW) 24 h after dehydration, and gradually decreased after rehydration (**Figure 1E**). Additionally, the activities of the antioxidant enzymes POD and SOD in D-R-treated gametophytes were also measured (**Figures 1F, G**). POD activity increased 2.1-fold compared with that in the control after 2 h of dehydration. SOD activity significantly increased to a peak after 6 h of dehydration (0.037 ± 0.0007 U gprot^-1^ DW). Upon rehydration, POD and SOD activity levels declined compared to the desiccation treatment. The POD activity was significantly higher than that of the controls at 0.5, 2, 6, and 24 h after rehydration; specifically, POD activity was 35.6%, 67.2%, 33.8%, and 75.8% higher in these rehydrated gametophytes than the control levels, respectively. Therefore, this increased expression allowed the plants to mount a robust antioxidant response, and recover to levels approaching the fully hydrated state (0 h) when rehydrated over 48 h (**Figures 1F, G**).

### Transcriptome assembly, assessment, and differentially expressed transcript analysis

**Figure 2:**
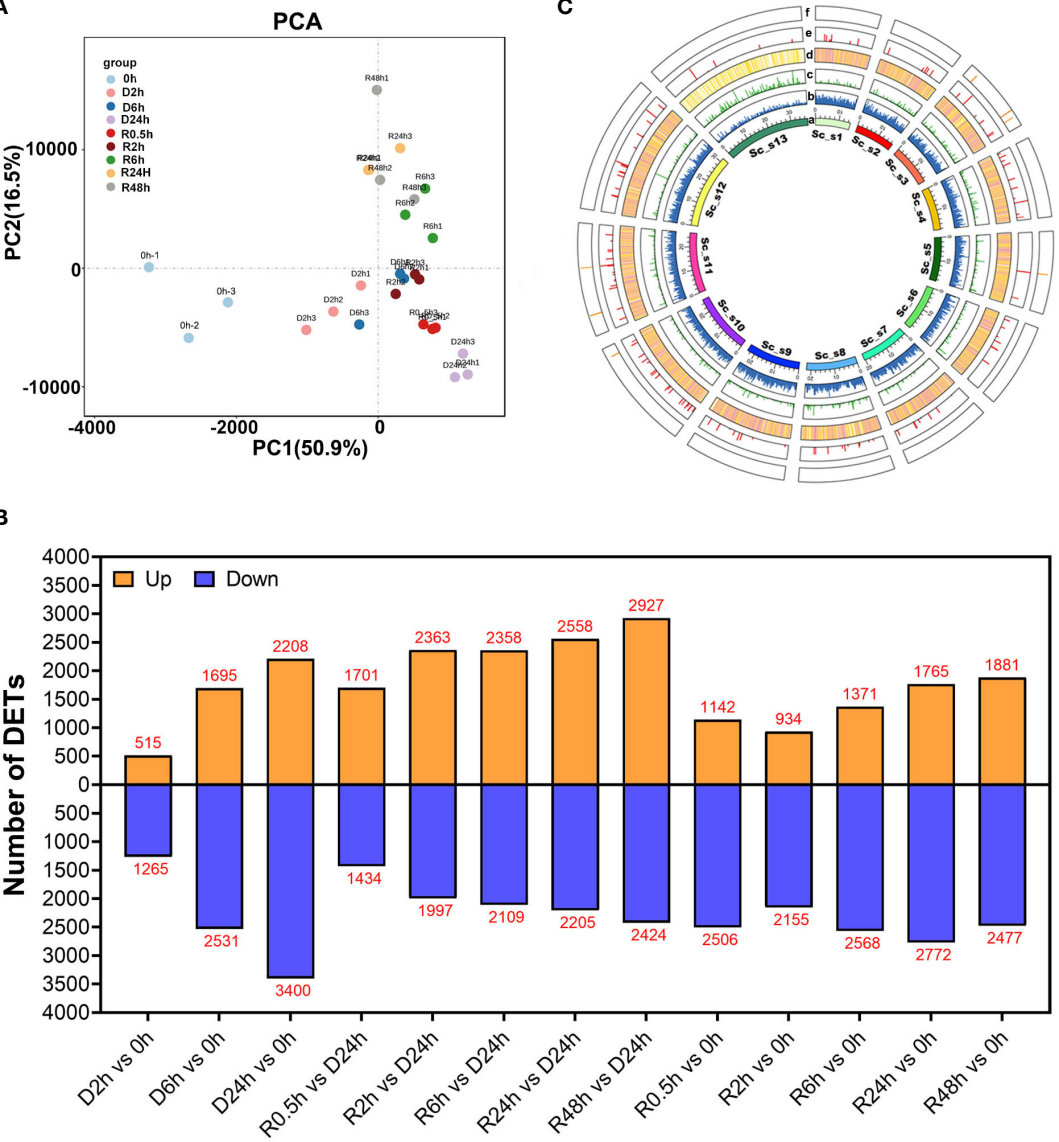
PCA, Circos, DETs of *S. caninervis* transcriptome during D-R treatment **(A)** PCA of RNA-Seq data obtained from *S. caninervis* of full hydrated (0 h), dehydrated (D h) and rehydrated (R h) gametophyte. **(C)** Circos visualization of genomic and transcriptomic features of *S. caninervis* under D-R process. a: Chromosomes; b: Gene distribution density, and the height of the column represents the number of genes at the within unit region; c: Novel transcripts distributions from transcriptome, the green column and length show its density mapping on chromosome; d: Transcript density distribution, the heatmap represent transcript density with white (min), yellow (middle) and red (max); e and f: Alternative splice position, the column of red and yellow represent SE and ME, the column of length represent their number of occurrences; **(B)** Number of DETs among different comparison pairs. |Log_2_ (Fold Change)| ≥1 and *p*-value ≤0.05 were used as the threshold to select DETs.

Transcriptome analysis of *S. caninervis* was performed using its corresponding genome as a reference during the D-R process. In the present study, nucleotide data in the transcriptome ranged from 5.08-9.92 Gb per the sequencing run in each treatment course (**Additional File 1**). After quality filtering, the number of clean reads was modified from 33-48×10^6^ in 27 samples (**Additional File 2**), which had an average read mapping rate of 80.86% for each sample on the *S. caninervis* reference genome. In total, 22489 transcripts were identified in the transcriptome of *S. caninervis*, of which 17152 transcripts could be mapped to the genome of *S. caninervis.* However, 5337 novel transcripts could not be mapped to the genome of *S. caninervis* (**Additional File 3**). Using principal component analysis (PCA) of *S. caninervis* transcriptomes, the treatment time points of D-R could be separated on the PCA axis, where PC1 explained 50.9% of the variation (**Figure 2A**).

In this study, the number of DETs was evaluated from 13 different comparison pairs (**Figure 2B**), including different D-R treatment time points (D/R h) compared to 0 h and rehydration treatment time points (R h) compared to D24h. Additionally, R h and 0 h comparison pairs were used to explore the changes in the transcripts of the moss after a round of desiccation compared to 0 h. Furthermore, R h compared to D24h was used to analyse the change in transcript differences between rehydration and absolute desiccation of D24h moss. During the dehydration process, 1810 DETs were in the D2h and 0 h comparison pair; this was the lowest number of DETs among all comparison pairs, of which 507 were up-regulated and 1203 were down-regulated transcripts. As dehydration progressed, the number of DETs gradually increased, with 4226 and 5608 DETs in comparison pairs D6h with 0 h and D24h with 0 h, respectively. Upon rehydration, the number of DETs exhibited a sharp decreasing trend in two comparison pairs (R0.5 h with 0 h and R2h with 0 h). Of the 3089 DETs detected in the R0.5h and 0 h comparison pair, 1142 transcripts were upregulated and 2506 transcripts were downregulated. Furthermore, the number of DETs increased gradually within the comparison pairs from R6h to R48h (**Figure 2B**). After a round of desiccation stress (R48h), the *S. caninervis* gametophytes were rehydrated; this process caused more differential changes in the number of transcripts than at 0 h (**Figure 2B**).

The common and specific DETs were analysed for each comparison pair, and 310 transcripts were found to be differentially abundant at all time points compared to that at 0 h (**Figure S1A**). Specifically, 47 transcripts were differentially abundant in all dehydration treatments compared with 0 h, while 46 were common in all rehydration treatments compared with 0 h (**Figure S1A**). There were 165 common DETs in the R h and D24h comparison pair (**Figure S1B**), and, overall, 19 DETs were present throughout all 13 different comparison pairs (**Figure S1C**).

We attached the transcripts obtained from the transcriptome sequencing to the chromosomes of the reference genome (**Figure 2C**). A total of 255 alternative splicing events were identified, of which only exon skips and mutually exclusive exons were detected 247 and 8 times, respectively.

### Transcriptome profiles of the gametophyte upon the D-R process

**Figure 3:**
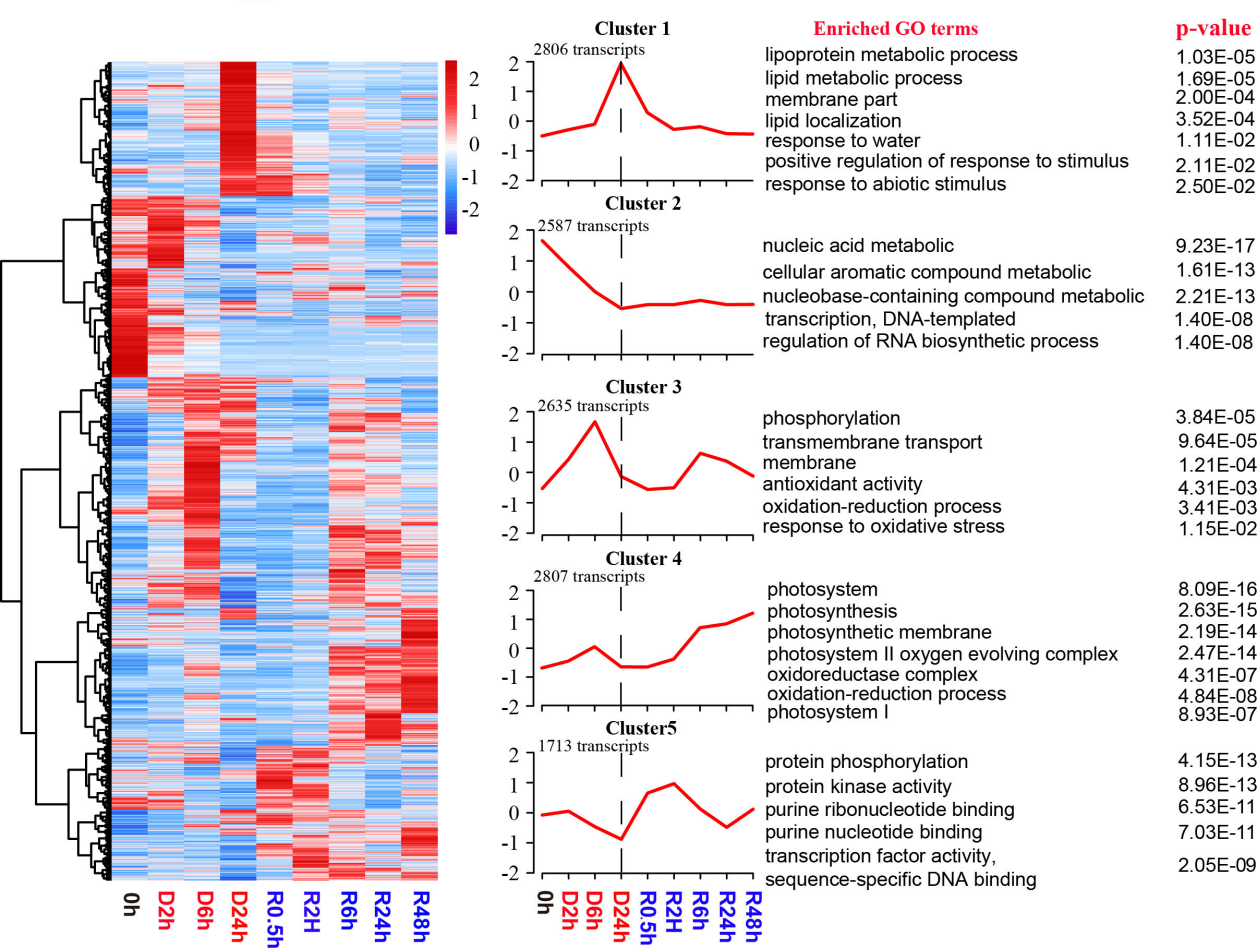
Global transcript abundance profiling of *S. caninervis* gametophyte during D-R process. |Log_2_(Fold Change)| ≥1 and p-value ≤ 0.05 were used as the threshold to select DETs; FPKM of DETs values are used for the heatmap, which up- and down-regulated are indicated in red and blue respectively; K-means clustering graph of DETs based on Z-score standardization of differential transcripts; DETs were clustered into five clusters by the K-means clustering (Cluster1-Cluster5). The number of transcripts in each cluster was showed at the top right-hand corner of each cluster. GO enrichment analysis of the five clusters and displaying significant GO terms with *p ≤*0.05.

A total of 12548 transcripts (representing 55.7% of protein-coding genes) exhibited significantly altered abundance levels (*p* ≤ 0.05) in the D-R samples in comparison to the abundance levels in the hydrated control samples (**Additional File 4**). To investigate the abundance profile of the transcriptome during the D-R process, K-means clustering analysis was performed on DETs to identify clusters with similar transcript abundance patterns. Five distinct clusters, named Cluster 1–5, were revealed; then, GO analysis was performed across the DETs from these 5 clusters (**Figure 3**; **Additional File 5**).

During desiccation stress, the transcripts were significantly accumulated into two clusters: Cluster 1 and Cluster 3. Cluster 1 contained 2806 transcripts. These Cluster 1 transcripts exhibited maximal abundance during the late stage of dehydration (D24h) and decreased immediately after re-watering. Additionally, this cluster was predominantly enriched in GO terms such as “lipoprotein metabolic”, “lipid metabolic process”, “membrane part”, “response to water”, “positive regulation of response to stimulus”, and “response to abiotic stimulus”. Cluster 3 showed two peaks in abundance at 6 h after early dehydration and 6 h after late rehydration. This cluster involved 2635 transcripts and was enriched in the GO terms “phosphorylation”, “membrane”, “membrane transport”, “antioxidant activity”, “oxidation-reduction process”, and “response to oxidative stress”. A total of 2587 transcripts were established within the Cluster 2 profile. The transcript abundances persistently declined during the whole desiccation process and maintained consistent transcript abundance levels in the late dehydration (D24h) and rehydration processes. Functional enrichment results demonstrated that the transcript abundance of Cluster 2 is associated with “nucleic acid metabolic”, “transcription, DNA-templated”, and “regulation of RNA biosynthetic process”. Alternatively, the transcript abundances of Cluster 4 continued to increase from 6 h to 48 h after rehydration and were primarily enriched in “photosystem I”, “photosystem II oxygen-evolving complex”, and “oxidation-reduction process”. This indicated that a large number of transcripts related to photosynthesis and antioxidation were transcribed after 6 hours of rehydration. In total, 1713 transcripts were observed within Cluster 5. These transcripts were significantly induced during the early rehydration period (R0.5h and R2h). Furthermore, the abundance of these transcripts decreased significantly in the late rehydration period (R6h-R48h). Transcripts in Cluster 5 were enriched in GO terms such as “protein phosphorylation”, “protein kinase activity”, and “transcription factor activity, sequence-specific DNA binding”.

### The KEGG pathways of DETs and WGCNA-trait analysis of desiccation tolerance

**Figure 4:**
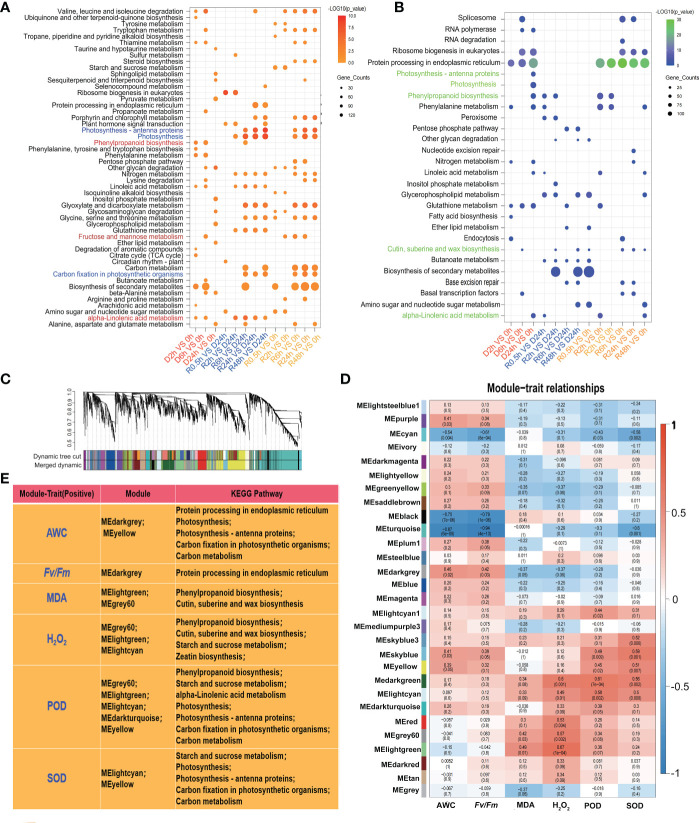
KEGG analysis and identification of modules related with the traits during D-R treatment in *S. caninervis.* **(A)** KEGG analysis of up-regulated DETs; Dots indicate the KEGG pathway with significant difference, and color indicates the degree of significance (From orange to red, the higher the significance) and the size of the circle indicates the number of transcripts. **(B)** KEGG analysis of down-regulated DETs; Dots indicate the KEGG pathway with significant difference, and color indicates the degree of significance (From blue to green, the higher the significance) and the size of the circle indicates the number of transcripts. **(C)** Transcripts dendrogram with cluster. Each module was assigned different colors. **(D)** Module-trait relationships. Each row corresponds to a colour module and column corresponds to a trait (AWC, *Fv/Fm*, MDA, H_2_O_2_, POD, SOD). Each block contains the correlation value and *p*-value; **(E)** KEGG analysis for screening positive correlation module with traits (AWC,*Fv/Fm*, MDA, H_2_O_2_, POD, SOD) and *p ≤* 0.05.

All upregulated and downregulated DETs in *S. caninervis* under D-R conditions were examined using the KEGG database. As shown in **Figure 4A**, the up-regulated DETs were enriched in various significant KEGG pathways, including “phenylpropanoid biosynthesis”, “alpha-linolenic acid metabolism”, “fructose and mannose metabolism”, “photosynthesis-antenna proteins”, and “carbon fixation in photosynthetic organism”. Among them, “phenylpropanoid biosynthesis”, “alpha-linolenic acid metabolism”, and “fructose and mannose metabolism” were enriched in the D2h with 0 h and D6h with 0 h comparison pairs, whereas the pathways related to photosynthesis were enriched after 6h of rehydration. Contrastingly, the downregulated DETs were enriched in “spiceosome”, “RNA polymerase, ribosome biogenesis in eukaryotes”, “protein processing in endoplasmic reticulum”, and “glutathione metabolism” (**Figure 4B**).

To better investigate the key transcripts which regulate desiccation stress responses in *S. caninervis*, FPKM of transcripts was used to construct WGCNA. The transcripts were divided into different gene sets (modules), and each module was assigned a different color (**Figure 4C**). The correlation between each module and six physiological indexes (AWC, *Fv/Fm*, MDA, H_2_O_2_, POD, and SOD) was assessed by plotting a heatmap of module-trait relationships (**Figure 4D**). As shown in **Figure 4E** and **Figure S2**, the AWC had a positive correlation with MEyellow (r = 0.39, *p* = 0.05) and MEdarkgrey modules (r = 0.46, *p* = 0.02); the transcripts of these two modules were enriched in KEGG pathways such as photosynthesis related pathways, “protein processing in endoplasmic reticulum”, and “phenylpropanoid biosynthesis”. The *Fv/Fm* values were positively associated with the MEdarkgrey module (r = 0.42, *p* = 0.03); these transcripts were enriched in the “protein processing in endoplasmic reticulum” KEGG pathways. MDA was positively correlated with two modules: MEgrey60 (r = 0.42, *p* = 0.03) and MElightgreen (r = 0.49, *p* = 0.01). H_2_O_2_ was positively correlated with 5 modules, of which MElightcyan (r = 0.49, *p* = 0.01), MElightgreen (r = 0.67, *p* = 1e-04), and MEgrey60 (r = 0.57, *p* = 0.002) were significant; the transcripts found in these modules were enriched in “phenylpropanoid biosynthesis”, “cutin, suberin, and wax biosynthesis”, “zeatin biosynthesis” and “starch and sucrose metabolism”. Moreover, POD and SOD activities had a strong correlation with MElightcyan, MEdarkturquose, MEyellow, MElightgreen, and MEgrey60; the transcripts in these modules were related to “starch and sucrose metabolism”, “alpha-linolenic acid metabolism”, “phenylpropanoid biosynthesis”, and photosynthesis related pathways.

### Molecular dynamics of pathways related to DT

**Figure 5:**
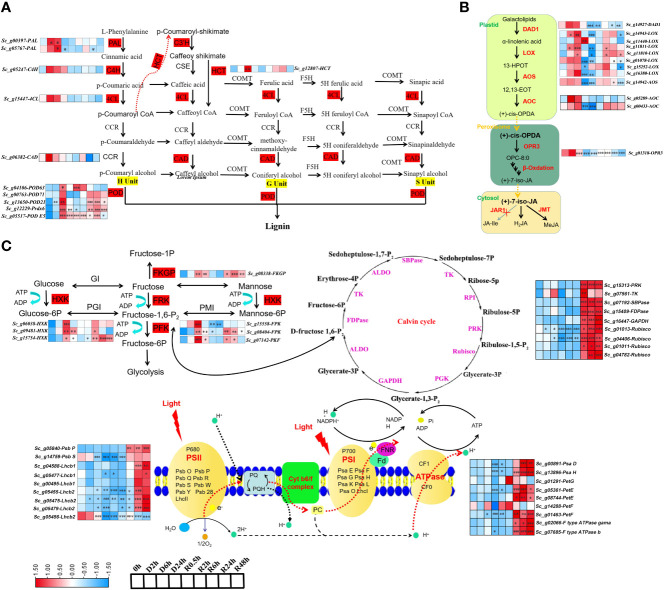
The dynamic change of transcript abundance in Key pathway during D-R process **(A)** alpha-linolenic acid; **(B)** phenylpropanoid biosynthesis; **(C)** fructose and mannose metabolism, photosynthesis and calvin cycle in *S. caninervis* during D-R process. DETs that up-and down-regulated are indicated in red and blue, respectively;FPKM values were used to heatmap; |Log_2_(fold change)|≥1 (*p<0.05, **p<0.01, ***p<0.001). Treatments during 24 h of desiccation (D h) and 48 h of rehydration (R h).

KEGG and WGCNA analyses revealed that the differentially expressed transcripts were enriched in phenylpropanoid biosynthesis, alpha-linolenic acid, fructose and mannose metabolism, photosynthesis, and carbon fixation-related pathways. Lignin biosynthesis, a major branch of phenylpropanoid metabolism, has been reported to play an important role in water deficit conditions (Sharma et al., 2019; Dong and Lin, 2021). The transcripts in lignin biosynthesis, including *ScPAL* (phenylalanine ammonia-lyase), *ScHCT* (*p*-hydroxycinnamoyl-CoA shikimate/quinate hydroxycinnamoyl transferase), and *ScPOD* (peroxidase) transcripts, were significantly expressed at 2 h and 6 h in the early stage of dehydration. Although not significant, *ScC4H* (cinnamate 4-hydroxylase), *Sc4CL* (4-coumaric acid: CoA ligase), and *ScCAD* (cinnamyl alcohol dehydrogenase) transcripts also accumulated during dehydration (**Figure 5A**).

Jasmonic acid (JA) is synthesised from alpha-linolenic acid, a major fatty acid in plant cell membranes. The DETs in this pathway, including *ScDAD1* (defective in anther dehiscence 1), *ScAOC* (allene oxide cyclase), and *ScLOX* (lipoxygenase), significantly increased in expression at 2 h and 6 h of dehydration compared with the control (0 h), and sharply decreased following 24 h of dehydration. The transcript abundance of *ScAOS* (allene oxide synthase) was stable in the early stage of dehydration from 0-6 h. Alternatively, the *ScOPR3* (12-oxophytodienoate reductase 3) transcript accumulated at 2 h and 6 h of dehydration, but was not significant compared with the control (0 h). Moreover, *ScLOX*, *ScAOC*, and *ScAOS* were induced during the rehydration stages (R6h, R24h, and R48h) (**Figure 5B**).

*ScHXK* (hexokinase), *ScFRK* (fructokinase), *ScPFK* (phosphofructokinase), and *ScFKGP* (L-fucokinase/GDP-L-fucose pyrophosphorylase), are key components in fructose and mannose metabolism. These transcripts significantly accumulated in the early stage of drying (6 h) and decreased after 24 h of dehydration (**Figure 5C**). Additionally, *S. caninervis* DETs involved in photosynthesis sharply declined in expression at 24 h of dehydration and significantly increased from 6-48 h of rehydration; these DETs included subunits of photosystems I and II (PSI and PSII) complexes (*ScPsa D*, *ScPsaH*, *ScPsbP*, and *ScPsbS*), photosynthetic electron transport (*ScPetG, ScPetE*, and *ScPetF*), F-type ATP synthase gamma/b, and the Calvin cycle (*ScRuBisCO* [rubisco ribulose-1, 5-bishosphate carboxylase], *ScTK* [transketolase], *ScRPI* [ribose 5-phosphate isomerase], *ScGAPDH* [glyceraldehyde-3-phosphate dehydrogenase], *ScFDPase* [fructose 1, 6-diphosphatase], and *ScRPK* [phosphoribulokinase]) (**Figure 5C**). Interestingly, 22 transcripts in the *ELIP* family and 45 *ScLEA* transcripts significantly accumulated in the late stage of dehydration (D24h) (**Figure S3**), indicating that they are important for the tolerance of late dehydration of *S. caninervis.* These results suggest that alteration of transcript abundance in these key pathways related to desiccation stress, such as *ELIP* and *LEA*, could contribute to improving the DT of *S. caninervis* during the different stages of D-R treatment.

### Differentially accumulated TFs under the D-R process

**Figure 6:**
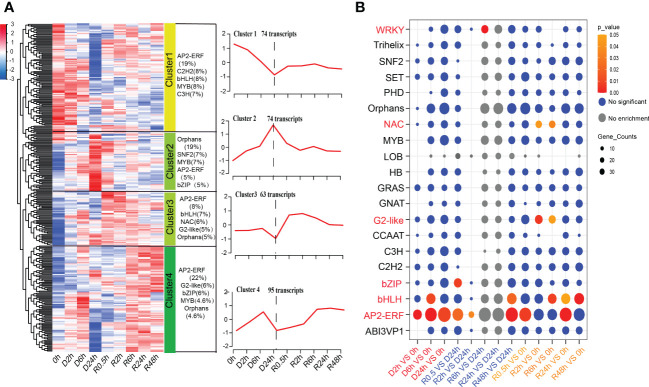
Global transcript abundance profiling and enrichment analysis of TFs during D-R process. **(A)** Heatmap and the dynamic accumulation patterns of the TFs; FPKM values are used for the heatmap and Clusters, (Cluster 1-Cluster 4) were generated by the K-means. The category and number of transcripts are marked in the upper part of each line chart. **(B)** Enrichment analysis of TFs in different comparison pairs. Circle size is correlated with number of TFs. TF family names are listed on the left, and different comparison pairs are listed on the bottom; red and orange circle represent *p ≤* 0.05, bule circle represent no significant, grey circle indicated no enrichment; Treatments during 24 h of desiccation (D h) and 48 h of rehydration (R h).

In the present study, the GO term enrichment of DETs in “transcription factor activity, sequence-specific DNA binding” indicated that TFs play important roles in the response of *S. caninervis* to DT (**Figure 3**). *AP2-ERF*, *bHLH*, *MYB*, and *C3H* were the largest groups of TFs during D-R treatments, accounting for 10%, 6%, 5%, and 5% of the total TFs, respectively. Differential analysis showed that 306 TFs were differentially altered during D-R treatment. These differentially abundant TFs belonged to 52 TF families. K-means clustering was performed on differential TFs to identify clusters with similar accumulated transcript patterns. Four distinct clusters, named Cluster 1-4, were identified (**Figure 6A**; **Additional File 6**). Clusters 1 and 2 both contained 74 differential TFs, of which Cluster 1 was downregulated and Cluster 2 was upregulated after 24 h of dehydration. The two clusters exhibited opposite responses during dehydration treatments. The top five TF families with the largest gene numbers in Cluster 2 were *Orphans*, *SNF2*, *MYB*, *AP2-ERF*, and *bZIP*, which constituted 19%, 7%, 7%, 5%, and 5% of Cluster 2 TF families, respectively. There were 63 differential TFs in Cluster 3, which showed little change within 6 h of dehydration and decreased to the lowest expression level at 24 h of dehydration. Additionally, this expression of Cluster 3 TFs increased rapidly after rehydration and gradually decreased to the control level (0 h) after 0.5 h of dehydration. Cluster 4 contained 95 differential TFs; the expression of these TFs reached a moderate peak after 6 h of dehydration and reached the lowest level after 24 h of dehydration. After re-watering, Cluster 4 transcript abundance continued to rise and reached an accumulation balance at 6 h of rehydration. The largest number of TFs in Clusters 3 and 4 were *AP2-ERF*, accounting for 8% and 22% of the total differential TFs in these clusters, respectively. Additionally, through TF enrichment analysis, we determined that *AP2-ERF*, *bHLH*, *G2-like*, *NAC*, *WRKY*, and *bZIP* were significantly enriched in the D-R process (**Figure 6B**). Of these, *WRKY*, *NAC*, and *G2-like* were enriched in the rehydration treatment, whereas *bZIP* was enriched only in the dehydration treatment. Furthermore, *AP2-ERF* and *bHLH* were strongly enriched in both the dehydration and rehydration treatments.

### Confirmation of DT-related transcriptional changes of represented DETs by RT-qPCR

**Figure 7:**
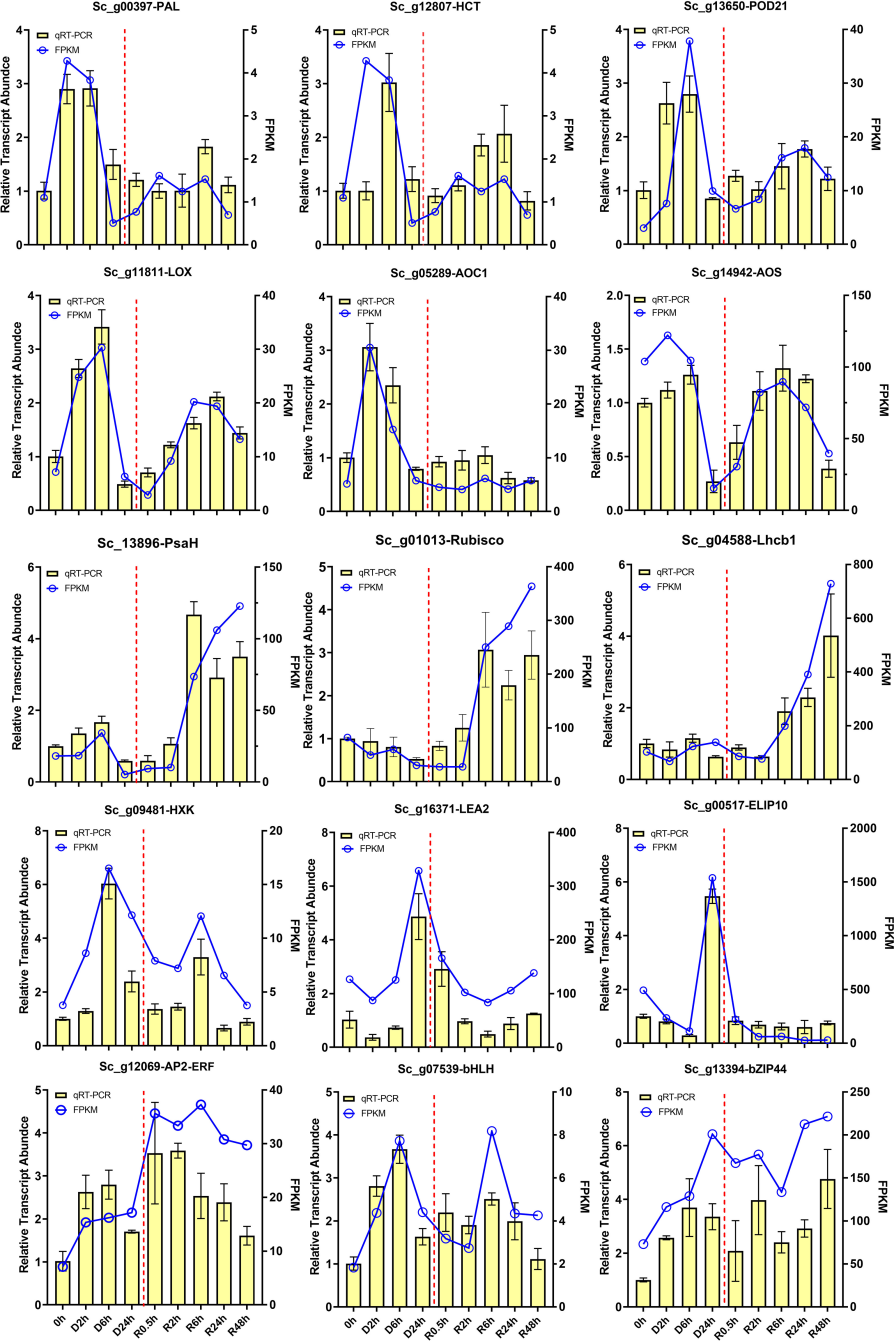
RT-qPCR validation of interested transcripts in *S. caninervis* during D-R process. Selected transcript abundance profiles were validated using RT-qPCR, including phenylpropanoid biosynthesis: *ScPAL* (Sc_g00397), *ScHCT* (Sc_g12807) and *ScPOD21* (Sc_g13650); α-linolenic acid pathway: *ScLOX* (Sc_g11811), *ScAOC1* (Sc_g05289) and *ScAOS* (Sc_g14942); photosynthesis and fructose and mannose metabolism: *ScPsaH* (Sc_g13896), *ScRubisco* (Sc_g01013), *ScLHcb1* (Sc_g04588), and *ScHXK* (Sc_g09481); DT-related trancripts: *ScLEA2* (Sc_g16371) and *ScELIP10* (Sc_g00517). TFs: *ScAP2-ERF* (Sc_g12069), *ScbHLH* (Sc_g07539), and *bZIP* (Sc_g13394). RT-qPCR data were showed as the mean ± SD. Line chart represents FPKM value, and bar chart represents RT-qPCR value.

To validate the RNA-seq results, we used RT-qPCR to analyse the transcript accumulation of 15 selected genes from four desiccation stress response KEGG pathways and two desiccation stress response marker genes, *LEA2* and *ELIP10* (**Figure 7**). The changes in the abundance of the selected DETs at different desiccation treatment points were in strong agreement with those of the RNA-seq results, indicating that transcriptome data accurately reflect *in vivo* transcript expression in the present study. The transcripts involved in phenylpropanoid biosynthesis, alpha-linolenic acid, and fructose and mannose metabolism pathways, including *ScPAL* (Sc_g00397), *ScHCT* (Sc_g12807), *ScPOD21* (Sc_g13650), *ScLOX* (Sc_g11811), *ScAOC1* (Sc_g05289), *ScAOS* (Sc_g14942), and *ScHXK* (Sc_g12069), were markedly upregulated in the early stage of D-R treatment. Furthermore, photosynthesis-related transcripts, such as *ScPsa H* (Sc_g13896), *ScRubisco* (Sc_g01013), and *ScLhcb1* (Sc_g04588), were significantly upregulated after 6 h of rehydration. Additionally, the abundance of three TFs, *ScAP2-ERF* (Sc_g12069), *ScbHLH* (Sc_g07539), and *ScbZIP* (Sc_g13394), significantly increased under both dehydration and rehydration conditions.

## Discussion

Bryophytes belong to basal plant lineages that first colonised land and have developed adaptive mechanisms to cope with desiccation stress. In the wild, the DT moss *S. caninervis* from the desert is often subjected to unpredictable D-R events; consequently, this moss species has evolved remarkable constitutive and inducible mechanisms of DT to survive in these adverse desiccation environments (Li et al., 2010). In contrast to vascular plants, particularly the model plant *Arabidopsis thaliana*, little is known about the water deficit responses in DT moss. Transcriptional profiles during the D-R process have been performed in DT moss, such as *T. ruralis* and *B. argenteum* (Wood et al., 1999; Gao et al., 2017). However, previous transcriptomes for desert mosses have limitations; these include gene annotations using genomes of other species and a low sequencing depth. Although our research group has previously generated a mixed transcriptome of *S. caninervis*, it was impossible to investigate detailed transcriptional dynamic changes. In this study, the D-R transcriptome of *S. caninervis* was optimised and improved; overall, this made our results more accurate and comprehensive. Our study obtained transcriptome data at nine time points, including one full hydration, three dehydration, and five rehydration time points. Further, we used the newly acquired genomic data from *S. caninervis* for gene annotation. These improvements in transcriptional analysis helped improve our understanding of the *S. caninervis* transcriptome, which allowed further exploration of the molecular mechanism of DT moss and resulted in the production of an extensive genetic resource.

### The positive effects of phenylpropanoid biosynthesis and alpha-linolenic acid pathways on the DT response of *S. caninervis* in the early stage of dehydration

The phenylpropanoid biosynthesis pathway is activated under drought stress, resulting in an accumulation of various phenolic compounds which have the potential to scavenge harmful ROS. This process results in the reduction of cell membrane peroxidation, thereby protecting plant cells from the destructive effects of oxidative stress (Sharma et al., 2019). Earlier transcriptomic studies carried out on DT plants, such as *B. argenteum*, *Myrothamnus flabellifolia*, and *Boea hygrometrica*, confirmed that the enhanced abundance of transcripts in the phenylpropanoid pathway help provide desiccation resistance (Ma et al., 2015; Xiao et al., 2015; Gao et al., 2017). In the current study, the peak abundance of phenylpropanoid pathway transcripts in *S. caninervis* occurred in the early stage of dehydration, aligning with the trend observed in *Myrothamnus flabellifolia* (Ma et al., 2015). Nonetheless, the main change in expression in the phenylpropanoid biosynthesis pathway of *S. caninervis* focused on lignin biosynthesis pathway. However, in other DT plants, such as *G. pilifera*, the flavonoid biosynthesis of phenylpropanoid is activated (Liu et al., 2022), suggesting that different DT plants have specific response pathways in phenylpropanoid biosynthesis.

Alpha-linolenic acid contributes to lipid metabolism as a strong antioxidant and as a precursor to the synthesis of JA, which acts as a phytohormone signalling molecule that stimulates the downstream anti-stress response (Creelman and Mullet, 1997; Wasternack and Feussner, 2018; Zi et al., 2022). JA is significantly induced in the early stages of dehydration in *H. rhodopensis* (Djilianov et al., 2013). Furthermore, *H. rhodopensis* desiccation stress transcripts were enriched in the early stages of dehydration (Liu et al., 2018). In addition, for *B. hygrometrica* and *Marchantia inflexa*, the GO terms of JA biosynthetic and signalling pathways have been observed to be enriched among the differentially abundant transcripts in response to dehydration (Zhu et al., 2015; Marks et al., 2021). In the present study, we found that these JA synthesis pathway transcripts in the *S. caninervis* transcriptome were significantly altered during the D-R process and elevated at the early stage of dehydration (2 and 6 h); this suggested that the JA synthesis pathway may have a positive effect on water-deficient stress in *S. caninervis.* Overall, these results indicate that the JA biosynthesis pathway may be an evolutionarily conserved response to DT, particularly in the early stages of desiccation response in DT plants.

### Photosynthesis pathway changes during the DT response of *S. caninervis*

Photosynthesis, which can be significantly affected by dehydration, is the most important photochemical reaction in plants (Challabathula et al., 2018). DT plants can reduce linear electron transport flux *via* the reorganisation of the photosynthetic apparatus, thus preventing oxidative stress (Liu et al., 2018). It has been observed that upon dehydration photosynthetic genes are downregulated, photosynthesis stops, and chloroplasts undergo reversible ultrastructural changes (Giarola and Bartels, 2015; Liu et al., 2018). In the DT moss *S. caninervis*, the abundance patterns of photosynthesis-related transcripts, such as PSI and PSII, light-harvesting complexes I and II, cytochrome b6/f complex, and electron transport proteins, were generally inhibited during dehydration treatments (**Figure 5C**); these findings are in agreement with other DT species, such as *C. plantagineum* (Giarola and Bartels, 2015) and *H. rhodopensis* (Liu et al., 2018). Interestingly, at the transcription level, the transcripts related to *S. caninervis* photosynthesis were not induced immediately after rehydration but were upregulated after 6 h of rehydration. This phenomenon may be caused by the fact that it is not necessary to synthesise all transcripts immediately during rehydration of *S. caninervis*; instead, this moss may be able to use mRNA stored in messenger ribonucleoproteins for initial protein translation (Wood and Oliver et al., 1999). Overall, at the transcriptional level, *S. caninervis* exhibited strong photoprotection capabilities during the dehydration process and rapid photosynthesis recovery during the rehydration process.

ELIP proteins are also important for photoprotection (Silva et al., 2021) and accumulate during the desiccation of many DT species (Zeng et al., 2002; Gechev et al., 2013; Yobi et al., 2017; VanBuren et al., 2019). LEA proteins are associated with drying plant tissues and play important roles in maintaining cellular integrity when water is reintroduced (Oliver et al., 2004). In the angiosperms, the *H. rhodopensis* and *C. plantagineum* expression levels of *LEA* transcripts increase upon desiccation stress (Piatkowski et al., 1990; Michel et al., 1994; Rodriguez et al., 2010; Gechev et al., 2013). Similarly, in the moss *P. patens* (Hiss et al., 2014) and *B. argenteum* (Gao et al., 2017), *LEA* transcripts are more abundant during the dehydration process. Additionally, in *S. caninervis*, *ELIP*, and *LEA* were determined to strongly accumulate following dehydration, especially during late-stage dehydration. Therefore, ELIP and LEA protein functions are conserved as key components in response to DT in both bryophytes and angiosperms.

### Desiccation tolerance related TFs in *S. caninervis*

TFs are important regulators involved in signal transduction and gene expression regulation under environmental stresses, such as water deficiency, temperature, salinity, and wounding stress (Singh and Laxmi, 2015; Yoon et al., 2020). In this study, desiccation stress caused differential changes in the abundance of 306 TF family members in *S. caninervis*. Among these TF families, 62 and 33 were associated with early dehydration and rehydration, respectively. In the DT moss *B. argenteum*, 404 TF-coding transcripts differentially accumulated during the time course of D-R. In total, 27 and 23 families were associated with dehydration and early rehydration, respectively (Gao et al., 2014). This indicates that *S. caninervis* possesses more TF families involved in DT than *B. argenteum*. Approximately 66% of differential TFs in *S. caninervis* were altered by desiccation stress. Specifically, seven key TF families (*AP2-ERF*, *WRKY*, *G2-like*, *MYB*, *bZIP*, *bHLH*, and *NAC*) were the most enriched families in D-R treatments of *S. caninervis*, indicating that these TFs are involved in the DT response. Similarly, in *B. argenteum*, *AP2-ERF*, *MYB*, *bZIP*, and *bHLH* were the most enriched TF families in response to D-R. AP2-ERF is a large family of plant TFs that play important roles in the control of plant metabolism and development, alongside various biotic and abiotic stress responses (Licausi et al., 2013). AP2-ERF is the largest TF family with the most differential abundant TFs in *P. patens* (Reboledo et al., 2022) and *B. argenteum* (Gao et al., 2017). Based on our transcriptome analysis, *AP2-ERF* was also the most abundant TF family in *S. caninervis* (Gao et al., 2014; Li et al., 2017). Many AP2-ERF family TFs are involved in the response of *S. caninervis* to DT and other biotic or abiotic stresses. For example, *ScDREB5*, *ScDREB8*, and *ScDREB10* have been observed to significantly improve drought and salt tolerance in *Arabidopsis* (Li et al., 2016; Li et al., 2019b). Additionally, *ScAPD1-like* enhances resistance to *Verticillium* wilt in transgenic *S. caninervis* and *Arabidopsis* (Li et al., 2023). Further, during the rehydration process in the current study, *NAC* transcript abundance rapidly and significantly increased in *S. caninervis*. NAC also contributes to water conduction in *P. patens* (Xu et al., 2014). Similarly, *NAC* in *Arabidopsis* regulates xylem vessel differentiation, thereby affecting water conduction. Previous reports have shown that *bHLH* genes isolated from angiosperms enhance the drought tolerance of plants (Gu et al., 2021; Qian et al., 2021; Gao et al., 2022). Specifically, *bHLH* is an important component in the stomatal development of *P. patens*, and is, therefore, involved in the regulation of drought tolerance (Caine et al., 2020). In our study, *bHLH* genes were significantly enriched in both the dehydration and rehydration processes. Our results, and those of previous studies, have shown that TFs play critical and evolutionarily conserved roles in the DT response of moss and angiosperms.

## Data availability statement

The dataset presented in the study are deposited in the China National GeneBank DataBase (CNGBdb) repository (https://db.cngb.org/), accession number CNP0003370.

## Author contributions

DZ, XL, and RY conceived the study. RY conducted the experiments and collected the data with the help of WB, YL, XJL and BG. RY performed all the analyses with the help of QY and MZ and wrote the manuscript. DZ and XL revised the manuscript. All authors contributed to the article and approved the submitted version.
