# Supplementary material for: Transcriptional profiling analysis providing insights into desiccation tolerance mechanisms of the desert moss Syntrichia caninervis
Source: Front Plant Sci. 2023 Feb 23;14:1127541. doi: 10.3389/fpls.2023.1127541 (PMC9995853; doi:10.3389/fpls.2023.1127541)
Supplement: Supplementary file 7 [file DataSheet_1.docx]

| Primer name | Primer sequence | Primer length | PCR amplification length |
| --- | --- | --- | --- |
| Sc_g00397-PAL F | GAGTGCTCGGAATGGATCGT | 20 | 110 |
| Sc_g00397-PAL R | AGCTGCAGAGCTTGATTGGT | 20 |  |
| Sc_g12807-HCT F | ACGCTGGTGGCGTTCTATC | 19 | 146 |
| Sc_g12807-HCT R | GGCTGAAAGTCCTCGGTTCT | 20 |  |
| Sc_g13650-POD21 F | AGCGAGTGCACTTCACCTAC | 20 | 147 |
| Sc_g13650-POD21 R | CCACATGCGGTTTGGTTCTG | 20 |  |
| Sc_g11811-LOX F | ATGAGCACCCGAACGAGTTT | 20 | 148 |
| Sc_g11811-LOX R | GCTGGGAGGTATGCCTTGTT | 20 |  |
| Sc_g05289-AOC1 F | ACGCTGTGTGTGTACGAGAT | 20 | 140 |
| Sc_g05289-AOC1 R | CCGTCGTAGAGCTTGTTGCT | 20 |  |
| Sc_g14942-AOS F | TACTTCTGGTTCCAGGGCGA | 20 | 134 |
| Sc_g14942-AOS R | TGGTCCAGCAAGCAGATCAC | 20 |  |
| Sc_g13896-Psa H F | CAGCGATACAACGGCTTCCA | 20 | 94 |
| Sc_g13896-Psa H R | CGCCTAGCACCAGAAACTTG | 20 |  |
| Sc_g01013-Rubsico F | CGCTACTGGACGATGTGGAA | 20 | 97 |
| Sc_g01013-Rubsico R | AGCTCGGGTACGTCTTCTTG | 20 |  |
| Sc_g04588-Lhcb1 F | GATGTTCGGGTTCTTCGTGC | 20 | 129 |
| Sc_g04588-Lhcb1 R | TAGTTGCCGGGGGTGAAGT | 19 |  |
| Sc_g09481-HXK F | ACGAGTTGGTCGGCAAGAAT | 20 | 114 |
| Sc_g09481-HXK R | ATAGCTTTGTGTGCCGGGAA | 20 |  |
| Sc_g16371-LEA2 F | CACCAAGGATATTGCGGTGC | 20 | 116 |
| Sc_g16371-LEA2 R | CGGAGAAATCATACGCCTGGT | 21 |  |
| Sc_g00517-ELIP10 F | CGGAAGAGCATGGAGACGAA | 20 | 150 |
| Sc_g00517-ELIP10 R | GAACACGCTCAGGAACGACT | 20 |  |
| Sc_g12069-AP2/EREBP F | ACTACAGTGCCACCACGAAC | 20 | 145 |
| Sc_g12069-AP2/EREBP R | TACGCGACATGGTACGAAGG | 20 |  |
| Sc_g07539-bHLH F | ACGTACAAGACCTGAAGCGG | 20 | 139 |
| Sc_g07539-bHLH R | TCACTCGTCCCAAAGCTGTC | 20 |  |
| Sc_g13394-bZIP44 F | GAGCCGAGAACACGCACATA | 20 | 93 |
| Sc_g13394-bZIP44 R | CGCTCGATCTCAGCACACAA | 20 |  |

Table S1 Primers information of qRT-PCR

Table S2 AWC and *Fv/Fm* of *S. caninervis* during desiccation (D h) and 48 h of rehydration (R h) for dehydration and rehydration treatment.

| Treatment (min or h) | AWC(g g-1 DW) | *Fv/Fm* |
| --- | --- | --- |
| 0h | 2.33±0.19 | 0.67±0.02 |
| D2h | 1.82±0.11* | 0.62±0.02** |
| D6h | 1.43±0.18** | 0.51±0.01*** |
| D24h | 0.045±0.02*** | 0±0*** |
| R5min | 1.80±0.06** | 0.52±0.02*** |
| R0.5h | 1.91±0.03* | 0.57±0.04* |
| R2h | 2.00±0.20 | 0.57±0.01** |
| R6h | 2.22±0.12 | 0.60±0.02** |
| R24h | 2.18±0.01 | 0.65±0.03 |
| R48h | 2.25±0.16 | 0.64±0.03 |

Data are presented as means ± SD, n=5. The data with asterisk shows that there are differences compared with the control (0 h) (*: *p* < 0.05, **: *p* < 0.01, ***: *p* < 0.001), as determined by analysis of variance (ANOVA) followed by LSD multiple comparison.


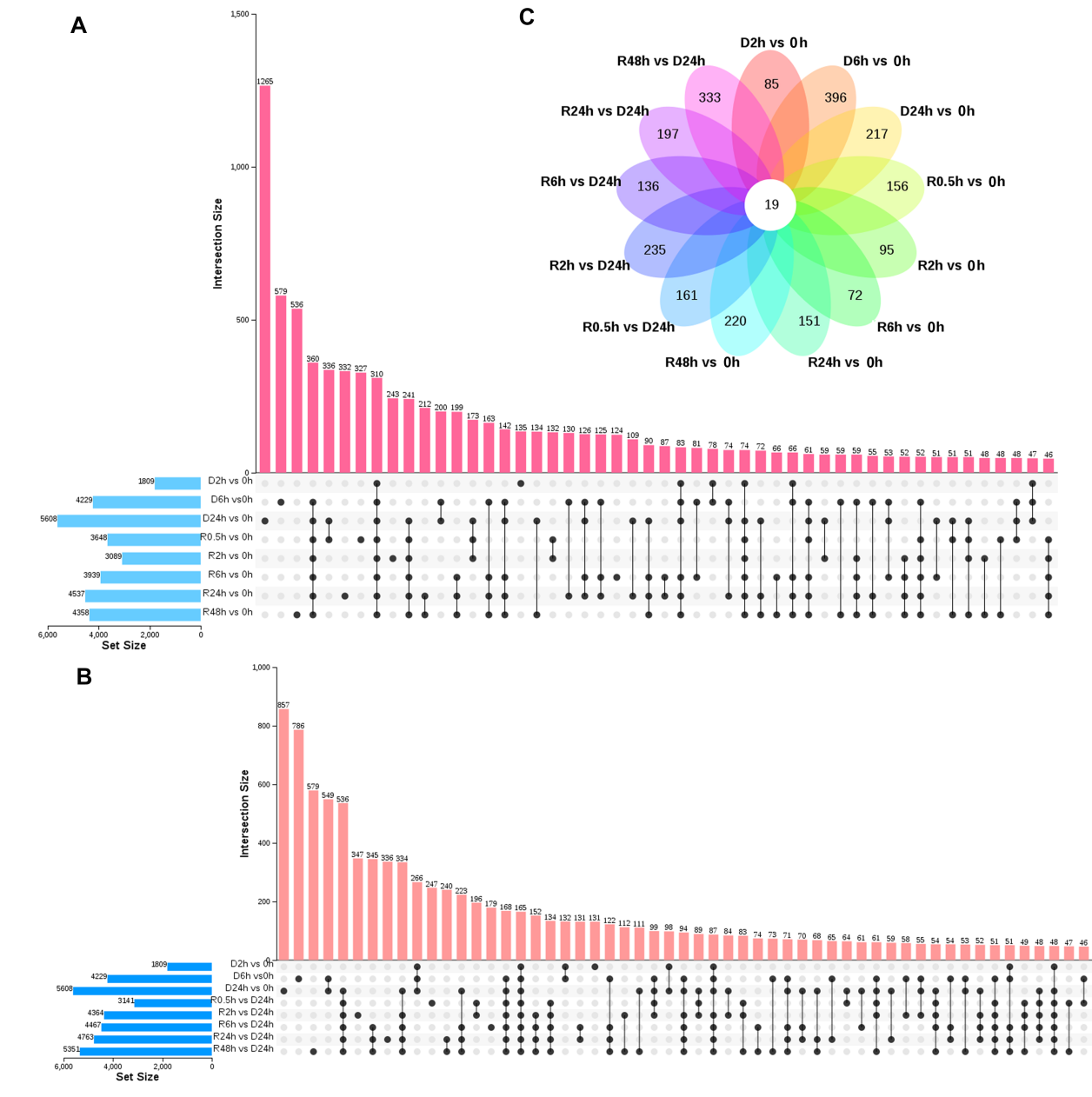


**Figure S1** Upset and flower plots of DETs among different comparison pairs of ***S. caninervis***. (A): Upset plot of DETs for dehydtation (D h) VS Full hydrated(0 h) and rehydtation (R h) vs Full hydrated(0 h); (B): Upset plot of DETs for dehydtation (D h) vs Full hydrated(0 h) and rehydtation (R h) vs Fully dehydrated state(D24h); (C): Flower plot of SDATs among different comparison pairs


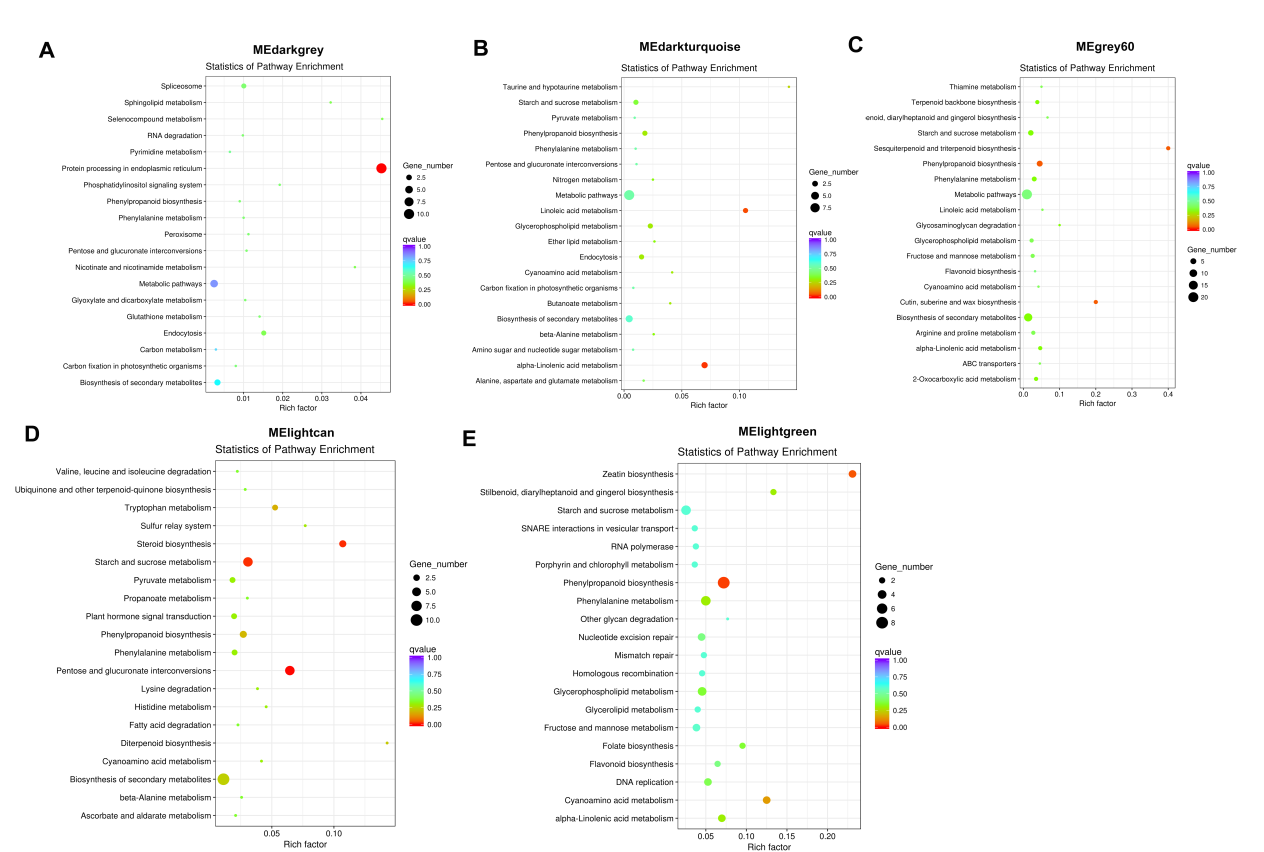


**Figure S2 KEGG analysis of transcripts in five module-trait modules of WGCNA. (A):MEdarkgrey module; (B): MEdarkturquoise module; (C):MEgrey60 module; (D):MElightcan (E):MElightgreen;** *p* ≤0.05 were used as the threshold to select significantly differentially KEGG pathways.The size of circle indicated the number of transcripts.


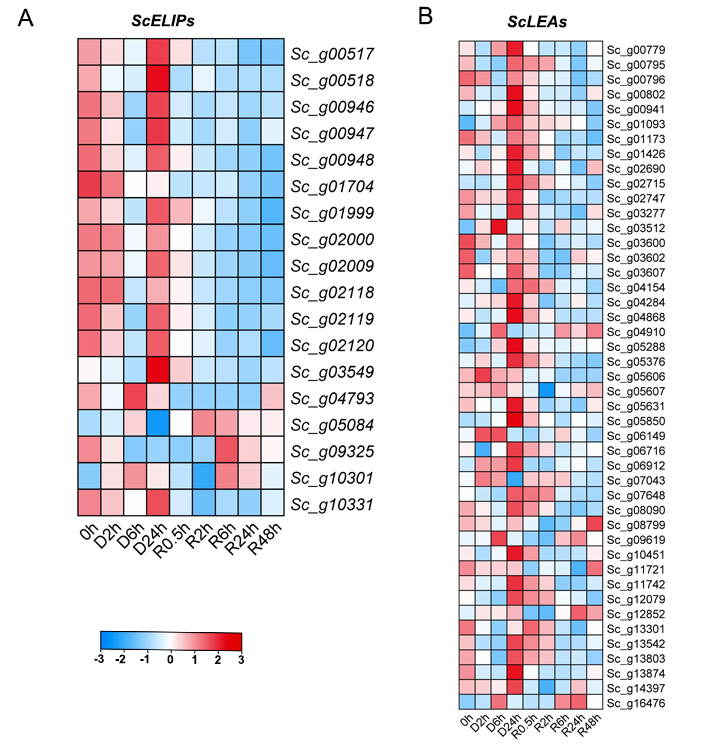


**Figure S3 Heatmap of transcripts abundance for *LEA* and *ELIP* families in *S. caninervis* during D-R process. The average of fragment per kilo base per million mapped reads (FPKM) values were used to heatmap; |Log_2_(fold change)|> 0（**p*<0.05, ***p*<0.01，****p*<0.001).** **Treatments during 24 h of desiccation (D h) and 48 h of rehydration (R h).**
